# Can They Make It on Their Own? Hosts, Microbes, and the Holobiont Niche

**DOI:** 10.3389/fmicb.2016.01647

**Published:** 2016-10-21

**Authors:** Sarah M. Kopac, Jonathan L. Klassen

**Affiliations:** Department of Molecular and Cell Biology, University of ConnecticutStorrs, CT, USA

**Keywords:** niche, symbiosis, selection, microbiome, reproductive rate, fitness, holobiont

## Abstract

Virtually all multicellular organisms host a community of symbionts composed of mutualistic, commensal, and pathogenic microbes, i.e., their microbiome. The mechanism of selection on host-microbe assemblages remains contentious, particularly regarding whether selection acts differently on hosts and their microbial symbionts. Here, we attempt to reconcile these viewpoints using a model that describes how hosts and their microbial symbionts alter each other's niche and thereby fitness. We describe how host-microbe interactions might change the shape of the host niche and/or reproductive rates within it, which are directly related to host fitness. A host may also alter the niche of a symbiotic microbe, although this depends on the extent to which that microbe is dependent on the host for reproduction. Finally, we provide a mathematical model to test whether interactions between hosts and microbes are necessary to describe the niche of either partner. Our synthesis highlights the phenotypic effects of host-microbe interactions while respecting the unique lifestyles of each partner, and thereby provides a unified framework to describe how selection might act on a host that is associated with its microbiome.

## Introduction

Virtually all multicellular organisms host a diverse collection of mutualistic, commensal, and/or pathogenic microbial symbionts, i.e., their microbiome (McFall-Ngai et al., 2013; note that we define “symbiosis” as “two organisms living together” regardless of the nature of this interaction, following De Bary, 1879). These symbiotic communities are assembled through vertical transfer between host generations and/or horizontal transfer from external environments, either neutrally or with host selection (Ebert, 2013). The long evolutionary association of hosts with microbes and the diverse phenotypes resulting from these interactions reinforce the broad influence of microbes on host fitness (McFall-Ngai et al., 2013). Despite this realization, how to best describe the evolution of hosts and their symbiotic microbes remains controversial.

One emerging paradigm to describe how hosts evolve alongside their microbial symbionts considers both hosts and their microbes as a single integrated unit, i.e., a holobiont (Zilber-Rosenberg and Rosenberg, 2008; Rosenberg and Zilber-Rosenberg, 2013; Bordenstein and Theis, 2015; Theis et al., 2016). Several aspects of this approach have been criticized, particularly the degree to which it presumes co-evolution between hosts and their microbes and whether selection on the holobiont supersedes selection on hosts and their symbionts individually (Moran and Sloan, 2015; Douglas and Werren, 2016). Although this debate remains unresolved, there is broad consensus that the impacts of microbial symbionts on host fitness need to be accounted for to comprehensively describe host evolution.

Models that describe how host fitness is modified by microbial symbioses are often rooted in evolutionary biology, perhaps reflecting a long history of co-evolutionary studies in host-microbe symbioses and proposed links to population genetics, e.g., via the “hologenome” (comprising both host and microbial genes; Zilber-Rosenberg and Rosenberg, 2008; Bordenstein and Theis, 2015). Here, we develop a complementary model rooted in ecological niche theory to describe how microbes alter host fitness. This framework advantageously accounts for the impacts of interactions between hosts and their microbial symbionts while respecting the distinct lifestyles and evolutionary interests of each partner. Based on this framework, we propose a mathematical model to determine if symbiotic microbes affect host fitness and illustrate experimental approaches that test this model. By accommodating the independent lifestyles of both hosts and their microbial partners while still allowing for emergent properties resulting from host-microbe interactions, niche-based models provide a useful framework to comprehensively describe host-microbe ecology and evolution.

## Interspecific interactions and the ecological niche

**Figure 1:**
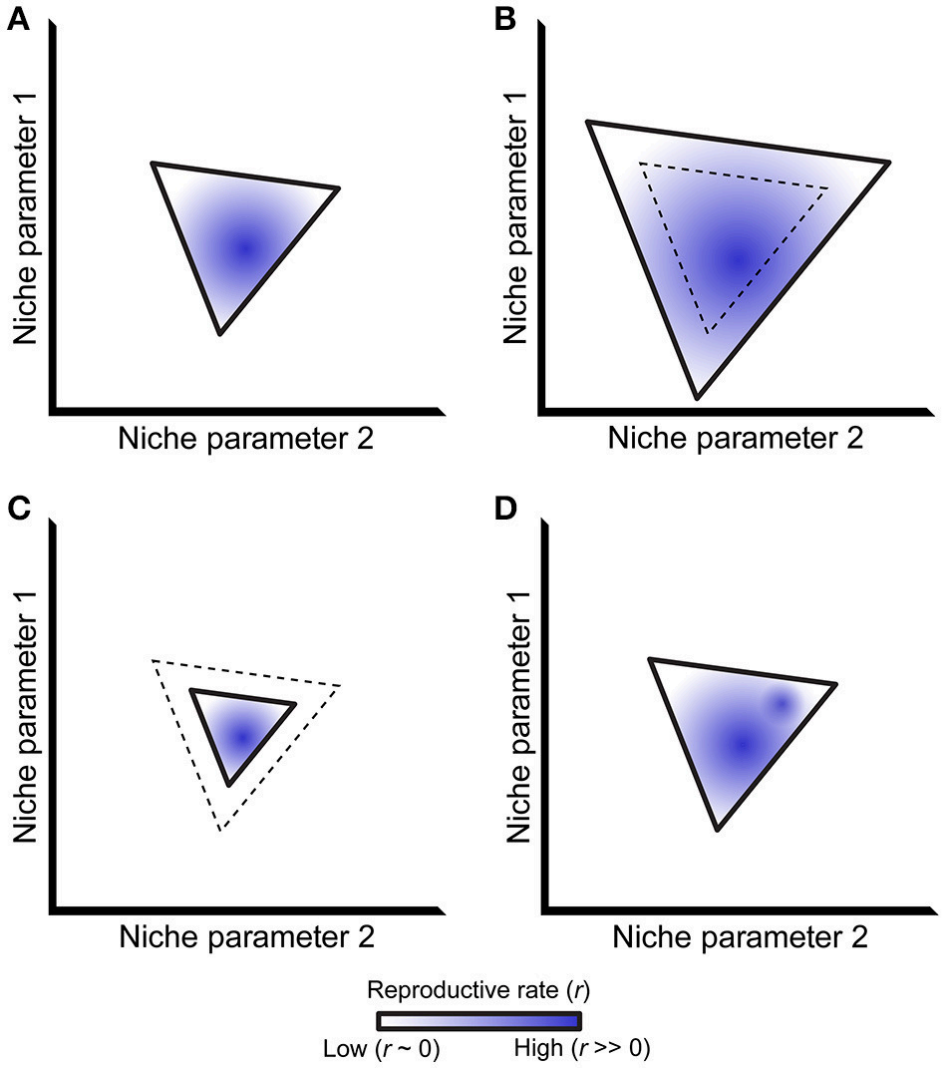
**The ecological niche and its relationship to reproductive rate**. **(A)** A simplified niche projected along two axes, bounded by a solid line representing the isocline where a species' net reproductive rate (*r*) equals 0. Stable population growth only occurs within this isocline, although net reproductive rates differ within this region as represented by different shades of blue. **(B-D)**: Niche boundaries may change relative to past conditions (dashed line) via expansion **(B)** or contraction **(C)**. The distribution of net reproductive rates may also change within a set of unchanging niche boundaries **(D)**, exemplified by the addition a second region of this niche having a high net reproductive rate. Although not shown in the figure, trade-offs may exist where niche boundaries both expand and contract in different parts of a species' niche alongside multiple changes in net reproductive rate.

In his landmark “Concluding Remarks” paper, Hutchinson defined a species' fundamental niche as the sum of all environmental factors that allow that species to reproduce and maintain a stable population over time (Hutchinson, 1957). Outside of this fundamental niche, the population death rate exceeds the population reproductive rate and, barring other forces, ultimately causes extinction. The fundamental niche can be represented as an n-dimensional vector that defines regions in niche space where a population will grow (*r* > 0, where *r* is the net reproductive rate), and regions where that population will ultimately go extinct (*r* < 0). The first two dimensions of such a vector are illustrated in Figure 1A, with solid lines representing the *r* = 0 isocline that separates the regions where *r* > 0 and *r* < 0. This isocline represents the boundary of a species' fundamental niche.

The fundamental niche represents an idealized situation without interspecific interactions. Accordingly, Hutchinson further defined the “realized niche” as a subset of the fundamental niche where a species is not outcompeted by other taxa (Hutchinson, 1957). Subsequent work has extended this idea to include mutualistic interactions that can expand the realized niche beyond the boundaries of the fundamental niche (Bruno et al., 2003). The structure of a host's realized niche therefore depends on the outcome of symbiotic interactions, including the entire symbiotic spectrum from pathogens to mutualists. It therefore follows that changes in such symbiotic interactions might change the structure of a species' realized niche (Figures 1B,C).

The realized niche describes where a species is likely to persist, having a net reproductive rate *r* > 0. Although a species will persist within this region, *r* will almost certainly not be equal throughout it. For example, different genotypes within a population will be better adapted to some parts of a niche but not others. Alternatively, some regions of the niche will remain incompletely filled because of dispersal limitation and/or low population density, and organisms will temporarily occur in regions where *r* < 0 due to source-sink dynamics (Pulliam, 2000; Holt, 2009). Thus, niche shape may change via differences in net reproduction rate, even within a constant niche boundary (defined by the *r* = 0 isocline; Figure 1D). Such changes in reproductive rate directly correspond to a species' evolutionary (Malthusian) fitness (Orr, 2009). In this way, understanding the shape of a species' niche and how it is altered by ecological interactions directly informs models of that species' fitness and evolution.

## Microbial modification of the host niche

The previous section describes how symbiotic microbes might alter the structure of the host niche, and thereby the host fitness landscape on which selection can act. Do such microbially-mediated niche alterations actually occur? Perhaps the most naïve test of this hypothesis is to compare microbe-free hosts to their colonized counterparts. These experiments often indicate that microbes modify the host niche, in the sense that growth rates and/or their proxies (e.g., development, disease susceptibility) differ between sterile and colonized hosts (Smith et al., 2007). However, such experiments cannot test if changes in the microbiota change the shape of the host niche because all hosts are colonized by microbes during birth, highlighting the artificiality of the gnotobiotic niche. Stronger evidence for microbially-mediated niche shifts comes from comparing natural host populations with different microbially-mediated phenotypes that effect host reproductive rate and/or related proxies. Ideally, these host phenotypes can be recapitulated in different host populations after switching microbes, thereby providing strong evidence that microbes alter the niches of their hosts.

Microbes can expand the niche boundary of many host species, as illustrated by Figure 1B. For example, defensive mutualists protect a host from infection by pathogens that would otherwise exclude it from the areas of its fundamental niche space containing those pathogens (reviewed in Oliver et al., 2014). Similarly, thermotolerance is conferred to the panic grass *Dichanthelium lanuginosum* via the fungus *Curvularia protuberata* when it is infected by the Curvularia thermal tolerance virus, thereby extending this grass's niche to include geothermally-heated soil (Márquez et al., 2007). This phenotype can be transferred to naïve tomato plants in cross-colonization experiments, highlighting the microbial etiology of thermotolerance (Rodriguez et al., 2008). Finally, many symbionts allow hosts to use substrates that they would otherwise be unable to live off of, e.g., plant sap lacking essential amino acids, animal blood lacking B vitamins, or inorganic ions emitted from deep-sea vents (Dubilier et al., 2008; Douglas, 2009). Niche expansion mediated by symbiotic microbes therefore occurs in many hosts and environmental contexts.

Microbes can also contract a host's niche, as illustrated by Figure 1C. An extreme example is pathogens that have driven their hosts extinct, such as malarial parasites of native Hawaiian birds (Warner, 1968; van Riper et al., 1986). Following the introduction of these pathogens, the realized niche of these hosts contracted to contain only pathogen-free regions. In another example, *Wolbachia* can cause cytoplasmic incompatibility in many insect hosts that prevent males from producing viable offspring with infected females (Werren, 1997), thereby reducing the infected host's niche to where uninfected mates are present. On a longer timescale, persistent associations with different nutritional mutualists have led to convergent losses of arginine biosynthetic genes in pea aphids (The International Aphid Genomics Consortium, 2010) and fungus growing ants (Nygaard et al., 2011; Suen et al., 2011), restricting these hosts to an obligately symbiotic lifestyle. This reflects a trade-off where niche expansion to use previously inaccessible nutrients was accompanied by niche contraction via diet restriction. Interestingly, microbes have also experienced niche contraction in these examples, e.g., via the loss of amino acid biosynthetic enzymes in *Buchnera* that leaves it dependent on its aphid host (Russell et al., 2013). Together, these examples demonstrate how niches can contract through parasitism and specialization.

The above examples highlight incidences where microbes change the host niche boundary, i.e., the *r* = 0 isocline (Figure 1). However, microbes may more often cause subtler changes in host net reproductive rate while leaving the niche boundary unaltered. For example, most pathogens do not drive their host extinct because pathogen fitness is dependent on successful transmission between infective and naïve hosts (Bull and Lauring, 2014). Instead, pathogen fitness is maximized when some but not all of their hosts are infected, i.e., host net reproductive rate is decreased but *r* > 0. In a second example, pea aphids reproduce twice as well on clover plants when infected with *Regiella insecticola* symbionts (Tsuchida et al., 2004), matching the occurrence of these *Regiella* only in regions where pea aphids colonized clover (Tsuchida et al., 2002). Thus, these aphids have increased fitness in a part of their niche that they occupy with *r* > 0 regardless of the presence of *Regiella*. Overall, we hypothesize that microbially-mediated changes in host net reproductive rates are more common than changes in host niche boundaries, especially for widely distributed species with broad environmental niches like humans. However, any change in niche shape can alter host evolution because of the direct relationship between net reproductive rate and Malthusian fitness (Orr, 2009).

## Host modification of the microbial niche

The preceding section describes how changes in a host's microbial symbionts can alter the host's realized niche. Might the reciprocal also be true, i.e., do associations with different hosts significantly alter the realized niche of a microbe? This depends on the fraction of a microbe's realized niche that depends on host association. At one extreme, obligate symbionts (e.g., *Buchnera* nutritional mutualists of aphids) lack any known life stage outside of their host; their realized niche is therefore entirely defined by host association (Douglas, 2009). In contrast, horizontally-transmitted microbes may replicate more frequently in non-host environments than host-associated ones (Mushegian and Ebert, 2016). For example, microbes that retain activity in the human gut after ingestion with food only briefly associate with hosts but reproduce extensively in food-associated niches (Derrien and van Hylckama Vlieg, 2015). Obligate symbionts and food-associated microbes represent extremes on a spectrum with many lifestyles in between. For example, *Xenorhabdus* symbionts of entomopathogenic *Steinernema* nematodes must balance carriage by the nematode against pathogenic potential toward prey insects (Chapuis et al., 2012). Such trade-offs are likely common for microbes that are horizontally transferred between hosts. In summary, the degree to which a host defines a microbe's niche is contingent upon on how much that microbe depends on that host to successfully replicate.

Even when hosts strongly define a microbe's realized niche, switching between different hosts may or may not shift this microbe's niche. For example, a generalist microbe may be active and equally adapted to multiple hosts, with equal reproductive rates in each. Alternatively, microbes may colonize non-target hosts (e.g., as zoonotic pathogens) in which they replicate poorly. Such colonization may be selectively favored if it is a byproduct of adaptations to another more common host, or selectively neutral if it happens at low frequency. Having said this, clear examples of horizontally-transferred microbes that are specialized to a particular host exist and represent instances where a specific host association dominates the structure of a microbe's niche (e.g., Kodaman et al., 2014). However, ecological forces such as drift and dispersal limitation must be ruled out as alternative explanations for such patterns of host-microbe specificity (Althoff et al., 2014). Explicitly differentiating between adaptive and non-adaptive reasons for host-microbe specialization is an important topic for future research.

## Selection on the host and its microbial symbionts

All hosts are colonized by microbial symbionts. Recent theories have suggested that both the host and its symbiotic microbes together form a single “holobiont” on which selection acts (Zilber-Rosenberg and Rosenberg, 2008; Rosenberg and Zilber-Rosenberg, 2013; Bordenstein and Theis, 2015; Theis et al., 2016). Expressed in terms of our niche-based framework, selection will follow the fitness landscape defined by the net host reproductive rates within its realized niche, as shaped by interactions with its microbial symbionts. The contributions of microbes to the host niche can therefore be formalized by the following equation:

(1) $$\begin{array}{l} N_{holo}=N_{apo}+A \end{array}$$

Here, a vector ***N*_*holo*_** describes the realized niche of the holobiont (holo- indicating whole, the host while associated with its microbial symbionts). This ***N*_*holo*_** vector can be decomposed into two other vectors, ***N*_*apo*_** describing the niche of the host lacking its microbiota (apo- indicating separate, the host without microbial symbionts) and ***A*** describing how ***N*_*apo*_** is modified by this microbiota. Experimentally, ***N*_*apo*_** can be observed as the niche occupied by a sterile host, and ***A*** represents how ***N*_*apo*_** differs from ***N*_*holo*_**. ***A*** can itself be decomposed into vectors describing the impact of individual members of a microbiome on the host niche and how interactions between these microbes alter the host niche. For example, gypsy moths are only sensitive to toxins produced by *Bacillus thuringiensis* when the moths are colonized by their natural gut microbes (Broderick et al., 2006). Thus, the interaction between *B. thuringiensis* and these gut microbes is required to describe how microbial symbionts together alter the moth's niche (as represented by ***A***). Equation (1) and its derivatives therefore mathematically describe how a host's realized niche is structured by interactions with its microbial symbionts, including emergent properties caused by interactions between multiple partners.

Equation (1) has several interesting properties. First, it provides an empirical measure to assess whether the holobiont might be a significant unit of selection according to the magnitude and shape of the host-microbe interaction vector ***A***. If selection acts along niche axes that are modified by ***A***, then selection on the host cannot be accurately represented without considering the influence of its symbiotic microbes. Thus, measuring the shape of ***A*** is a critical test of the holobiont as a unit on which selection might act. For example, differences between axenic hosts and those colonized by their microbes would define the value of ***A*** and indicate that microbes alter the shape of the host niche. Importantly, this vector describes changes in host net reproductive rate that are caused by microbes, which directly relates to host fitness. The ability of selection to maximize holobiont fitness must also be weighed against other ecological forces that impact reproductive rates within a niche, e.g., population density and dispersal rates.

Second, equation (1) explicitly describes changes to the niche of a single organism. Although holobiont theory was originally described in terms of selection on the host (Zilber-Rosenberg and Rosenberg, 2008; Rosenberg and Zilber-Rosenberg, 2013; Bordenstein and Theis, 2015; Theis et al., 2016), equation (1) can equally be written to describe how a microbe's niche is modified by the host. Differences between host and microbial generation times, population sizes, and life-cycle stages imply both that hosts and their microbial symbionts have differently shaped niches, and that the extent to which a microbe alters a host's niche (***A*_*microbe*_**) differs from the extent to which a host alters a microbe's niche (***A*_*host*_**). For example, horizontally-acquired microbes that reproduce more frequently in non-host environments than while host-associated (Mushegian and Ebert, 2016) likely have niches that remain unaltered by their host (***A*_*host*_** ≈ 0) despite these microbes' potential to impact their host's niche (***A*_*microbe*_** > 0). Members of the microbiome may also differ in their impact on their host's niche, either individually or as a community. Comparing ***A*** for different members of the microbiome and/or ***A*_*host*_** may be useful to identify shared environmental niches and/or coevolution between these partners.

Third, equation (1) can be easily expanded to describe how changes in a host's symbiotic microbes will change the niche of the holobiont:

(2) $$\begin{array}{l} \Delta N_{holo}=\;\Delta N_{apo}+\;\Delta A \end{array}$$

Given that hosts have been colonized by microbes throughout their evolutionary history, this is likely the most relevant question to ask concerning host evolution. Equation (2) suggests an important experimental approach to determine how microbes change the shape of the host niche. Microbially-mediated alterations of the holobiont niche shape can be determined by comparing the niche shape of a host species with different microbial symbionts or vice versa. This approach has already been applied in gnotobiotic experiments to determine the microbial etiology of different phenotypes exhibited by the same host genotype, i.e., where Δ***N*_*apo*_** = 0 (Smith et al., 2007). Similar exchanges using distinct host populations will allow disambiguation of host and microbial contributions to changes in a host population's niche shape, as exemplified by experiments describing how microbes facilitate diet specialization via toxin degradation in different populations of *Neotoma* woodrats (Kohl and Dearing, 2012; Kohl et al., 2014).

Finnaly, the notation “holo” to describe the host plus its microbes and “apo” to describe the host alone draws a direct parallel to the well-established nomenclature of “holoenzyme” and “apoenzyme” in biochemistry. Here, a holoenzyme comprises both an enzyme and its catalytic cofactor that together perform a biochemical function. In contrast, an apoenzyme lacks this cofactor and does not perform the biochemical function. Like hosts and their microbes, enzymes and their cofactors have different evolutionary capacities and/or tendencies, e.g., inorganic cofactors cannot evolve. Furthermore, the holoenzyme is not always the most relevant unit to describe biochemical function. For example, human cytosolic aconitase has enzymatic activity at high iron concentrations, but disassembles its cofactor at low iron concentrations. At such low-iron concentrations, this aconitase instead functions as an iron-responsive element binding protein that stabilizes ferritin mRNA and thereby increases its translation and ultimately iron accumulation (Kennedy et al., 1992). Such “moonlighting proteins” are common throughout biochemistry (Jeffery, 1999). Thus, like the holobiont, whether the holoenzyme is a relevant functional unit depends on whether or not the process of interest is affected by the presence of the partner cofactor.

In summary, many cases exist where a host's realized niche is altered by the presence of its microbial symbionts. These alterations can either increase or decrease the extent of the host's realized niche and reproductive rates within it, thereby altering host fitness as the target of selection. Such a niche shift can be identified by comparing sterile and colonized hosts (Smith et al., 2007), and/or using gnotobiotic systems that can experimentally differentiate host- and microbially-mediated alterations of niche shape (Kohl and Dearing, 2012; Kohl et al., 2014). A host might similarly shift a microbe's niche, although the strength and/or direction of such changes likely differ between these partners. We therefore consider hosts and microbes to comprise a single evolutionary unit in the sense that both partners may be required to faithfully describe their respective realized niches, and thereby the context in which selection can act.

## Author contributions

SK and JK both conceived, wrote, and edited the manuscript. All authors have approved the final manuscript version.

## Funding

This work was supported by funding to JK from the University of Connecticut.

### Conflict of interest statement

The authors declare that the research was conducted in the absence of any commercial or financial relationships that could be construed as a potential conflict of interest.
